# GSC State Plasticity Drives Therapeutic Resistance and Recurrence in Glioblastoma

**DOI:** 10.3390/cells15191757

**Published:** 2026-09-27

**Authors:** Jinxi Shuai, Cuicui Chang, Yanju Gong

**Affiliations:** 1 Chengdu University of Traditional Chinese Medicine, Chengdu 611137, China; 2 School of Clinical Medicine, Chengdu University of Traditional Chinese Medicine, Chengdu 611137, China; 3 School of Basic Medical Sciences, Chengdu University of Traditional Chinese Medicine, Chengdu 611137, China

**Keywords:** glioblastoma, glioblastoma stem cells (GSCs), state plasticity, therapeutic resistance, tumor recurrence

## Abstract

Glioblastoma (GBM) is the most aggressive primary brain tumor in adults, and treatment failure is closely linked to the presence of GBM stem cells (GSCs). Rather than representing a fixed marker-defined state, GSC identity is better understood as a dynamic and reversible stem-like state. GBM cells exhibit transcriptional plasticity, most clearly reflected by the proneural-to-mesenchymal transition, although direct interconversion among neural progenitor cell-like, oligodendrocyte precursor cell-like, astrocyte-like, and mesenchymal-like states remains unproven. GBM cells can also transition bidirectionally between stem-like and differentiated phenotypes, shift between quiescent and proliferative states, and reprogram metabolism in response to nutrient deprivation, hypoxia, and therapy. Perivascular, hypoxic, extracellular matrix, and immunosuppressive niches further regulate these transitions. This plasticity contributes to therapeutic resistance because radiotherapy, temozolomide, and targeted agents can both select preexisting tolerant populations and induce adaptive states. Therapy-persistent GSC-like cells may form residual reservoirs that undergo further remodeling and contribute to recurrence. Emerging strategies, most still preclinical, aim to destabilize stem-like states, broaden coverage across heterogeneous GSC populations and protective niches and block therapy-induced adaptive transitions. Thus, constraining GSC state plasticity may provide a framework for overcoming therapeutic resistance and recurrence in GBM.

## 1. Introduction

Glioblastoma (GBM) is the most common and aggressive primary malignant brain tumor in adults [1]. Despite maximal safe resection followed by radiotherapy plus concomitant and adjuvant temozolomide (TMZ) [2], the prognosis of GBM remains poor, with a median survival period of less than two years and almost definite recurrence [3]. Treatment failure is closely related to the presence of GBM stem cells (GSCs) [4], which are operationally characterized by stem-like properties, including self-renewal, tumor-initiating capacity, and the ability to recapitulate tumor heterogeneity [5]. By prioritizing the activation of DNA damage responses, GSCs develop resistance to radiotherapy and chemotherapy [6], making them core drivers of treatment resistance and tumor recurrence.

Importantly, GSC identity does not reflect a fixed and uniform state but instead reflects dynamic and reversible stem-like states shaped by intrinsic pathways and environmental signals [7]. Malignant GBM cells can occupy neural progenitor cell-like (NPC-like), oligodendrocyte precursor cell-like (OPC-like), astrocyte-like (AC-like), and mesenchymal-like (MES-like) transcriptional states [8,9] that are reshaped by microenvironmental and therapeutic pressures [10]. Stem-like and differentiated GBM compartments are dynamically connected, whereas experimentally defined GSC populations can shift between quiescent and proliferative states [11,12] and undergo metabolic reprogramming in response to nutrient deprivation, hypoxia, and treatment [13,14]. Perivascular, hypoxic, extracellular-matrix, and immunosuppressive niches provide spatially organized signals that regulate these transitions between states [15,16,17]. GSC identity is thus continuously remodeled across molecular, metabolic, and spatial dimensions.

Therapeutic pressure can both select preexisting stem-like states and induce new GSC-like states [18]. Indeed, radiotherapy, TMZ, and targeted agents can each drive adaptive state transitions that confer reversible drug tolerance [19,20,21]. Quiescent GSCs can survive therapy and later reactivate to contribute to tumor recurrence [22,23], whereas residual stem-like populations undergo further state remodeling during recurrent tumor reconstruction [24,25]. GSC plasticity thus links the biological properties of stem-like cells to the clinical course of GBM and is essential for explaining why current therapies fail.

To our knowledge, although GSC plasticity has been reviewed from several perspectives, its transcriptional, metabolic, and niche-regulated dimensions have rarely been integrated within a unified framework. In this review, we summarize the current understanding of GSC state plasticity, examine how it contributes to therapeutic resistance and recurrence, and discuss emerging strategies to therapeutically constrain this plasticity.

## 2. Defining GSC Identity

### 2.1. Cells of Origin Versus Those in GSC-like States

GBM has no confirmed cell of origin [26]. Human neural stem cells (NSCs), glial progenitor cells (GPCs), and OPCs, which carry specific carcinogenic alterations, generate migratory glioma-like populations in the mouse brain, whereas cell lineages and driver gene mutations together shape tumor heterogeneity [27]. Subventricular zone (SVZ) NSCs and OPCs are considered the leading candidate cells of origin for GBM, whereas mature glia may undergo oncogenic dedifferentiation.

GSC-like states are dynamic and reversible, allowing differentiated tumor cells to regain stem-like features under microenvironmental or therapeutic stress [28]. Cells occupying these stem-like states may subsequently contribute to the reconstitution of tumor heterogeneity and recurrence [28,29]. Notably, GSC-like states emerge during tumor evolution and should not be equated with the cells of origin of GBM [26]. Therefore, GBM may arise from multiple neural or glial cell types, whereas GSC-like states emerge during tumor evolution and contribute to tumor heterogeneity, treatment resistance, and recurrence.

### 2.2. GSC Markers and Their Limitations

Key GSC-associated markers include cluster of differentiation 133 (CD133), cluster of differentiation 44 (CD44), cluster of differentiation 15 (CD15), Nestin, SRY-box transcription factor 2 (SOX2), and Musashi-1 [30]. GSC-associated markers lack universality; specifically, cluster of differentiation 24 (CD24), CD133, epidermal growth factor receptor (EGFR), and CD44 preferentially mark NPC-like, OPC-like, AC-like, and MES-like states, respectively, indicating that these markers are more suitable for state stratification than for universal GSC identification. Experimentally defined GSC populations should instead be validated functionally through self-renewal, tumor initiation upon serial transplantation, and recapitulation of tumor heterogeneity [31,32]. Additionally, partitioning-defective protein 6 (Par6)/SOX2 modulation alters stemness marker expression and GSC sphere formation, indicating that marker levels vary with the state of stemness [33]. Marker-defined stem-like subsets differ in treatment sensitivity; for example, compared with CD133^−^ tumor cells, CD133^+^ glioma cells are more radioresistant, whereas treatment may increase the number of resistant stem-like subsets that contribute to recurrence [34]. Overall, functional assays with context-dependent marker profiles that reflect dynamic cellular states and treatment responses should be integrated into GSC identification, thereby increasing diagnostic accuracy.

## 3. State Plasticity of the GSC

### 3.1. Transcriptional Plasticity of GSC-like States

GSC-like tumor cells exhibit four major transcriptional states, namely, NPC-like, OPC-like, AC-like, and MES-like states, and their relative proportions can be reshaped by the microenvironment and therapeutic stress [35,36]. The most clearly characterized transformation is the stress-induced transition from the proneural pathway widely associated with NPC-like and OPC-like states to the MES-like state [37]. This transition illustrates the dynamic remodeling of GSC-like states, although direct interconversion among all four states remains unproven.

### 3.2. Bidirectional Transitions Between Stem-like and Differentiated States

The stem-like state is not confined to a preexisting cell population, as differentiated GBM cells can regain their stem-like properties under therapeutic stress [38]. TMZ induces this transition through senescence-associated reprogramming, whereas radiotherapy promotes it through metabolic reprogramming and carbonic anhydrase IX (CA9)-dependent extracellular acidification [39,40]. Together, these findings indicate that differentiated GBM cells retain treatment-responsive plasticity and can enter a GSC-like state through therapy-specific reprogramming mechanisms.

Conversely, state plasticity is bidirectional, with differentiated GBM cells reacquiring stemness and cells occupying GSC-like states exiting the stem-like state to adopt distinct cellular fates in response to intrinsic and microenvironmental cues [41]. For example, synapsin III (SYN3) promotes the neuronal-like transdifferentiation of GSCs by disrupting jagged canonical Notch ligand 1 (JAG1)-Notch receptor 1 (NOTCH1) interaction, whereas under hypoxia, tumor-associated macrophage (TAM)-derived Wnt family member 5A (WNT5A) activates receptor tyrosine kinase-like orphan receptor 1 (ROR1) signaling to drive the transdifferentiation of GSCs into glioma-derived endothelial cells [42,43]. Thus, GSC lineage plasticity can either reduce stemness or promote vascular remodeling, depending on the differentiation route.

### 3.3. GSC Quiescence-Proliferation Plasticity

Quiescence within GSC populations represents a prolonged but reversible cell cycle state associated with stress resistance and therapeutic resistance [44]. In glioma models, stress-adaptive complement C5a receptor 1 (C5AR1) signaling promotes G0 maintenance, whereas activation of mitogenic EGFR-adrenomedullin (ADM)-signal transducer and activator of transcription 3 (STAT3) signaling promotes GSC self-renewal and tumor growth [45,46]. Following chemotherapy, surviving coagulation factor III (F3; tissue factor)-^+^ quiescent GSCs become activated and transition toward proliferation, contributing to tumor expansion and recurrence [47]. In mouse GSC models, leucine-rich repeats and immunoglobulin-like domains 1 (Lrig1) regulate the balance between quiescence and proliferation, with its loss promoting proliferation and its overexpression enhancing quiescence [48]. The balance between quiescence and proliferation in GSCs is dynamically regulated by intrinsic signaling and therapeutic stress.

## 4. GSC Metabolic Plasticity

### 4.1. Baseline Metabolic Heterogeneity in GSCs

GBM exhibits substantial baseline metabolic heterogeneity, whereas experimentally defined GSC models exhibit additional state-dependent metabolic differences. Patient-derived GBM models exhibit distinct glycolytic and oxidative metabolic phenotypes, indicating substantial cell-intrinsic metabolic heterogeneity [49]. Metabolic features also vary according to cellular identity and state. Compared with their differentiated counterparts, GSCs present enhanced malate-aspartate shuttle activity [50]. In GSC models, dihydropyrimidine dehydrogenase (DPYD) expression is associated with mesenchymal features, suggesting state-dependent differences in pyrimidine metabolism [51]. Together, these findings indicate that both broader GBM populations and experimentally defined GSC models can occupy distinct metabolic configurations associated with cellular identity, stemness, and transcriptional state, representing the metabolic landscape on which adaptive plasticity can operate.

### 4.2. Stress-Induced Metabolic Reprogramming in GSCs

Beyond this baseline heterogeneity, GSCs dynamically reprogram their metabolism in response to changing microenvironmental and therapeutic conditions. Under glucose deprivation, patient-derived GSCs undergo subtype-dependent reconfiguration of endoplasmic reticulum (ER)–mitochondrial communication and bioenergetics, supporting metabolic flexibility under nutrient stress [52]. When glutamine availability is limited, GSC survival becomes increasingly dependent on pyruvate carboxylation to sustain metabolic fitness [53]. Under hypoxia, GSCs activate galectin-8 (Gal-8)-mechanistic target of rapamycin (mTOR)-transcription factor EB (TFEB)-dependent autophagy to sustain survival and self-renewal under oxygen-limited conditions [54]. Under irradiation, GSCs rapidly reprogram glycolytic flux toward phosphoglycerate dehydrogenase (PHGDH)-dependent serine synthesis to support DNA repair and radioresistance [55]. TMZ-induced glucose-6-phosphate isomerase (GPI) inactivation switches glucose flux from glycolysis toward the pentose phosphate pathway, promoting chemoresistance in GSCs [56]. These studies demonstrate that metabolic plasticity of GSCs enables adaptation to nutrient, hypoxic, and therapeutic stress by preserving bioenergetic flexibility, metabolic homeostasis, and biosynthetic capacity, thereby supporting survival and treatment resistance (Figure 1).

## 5. Niche Regulation of GSC State Plasticity

Spatially and functionally heterogeneous niches provide distinct microenvironmental contexts that shape the maintenance and adaptation of GSC-associated states. Recent spatial and single-cell studies have demonstrated that malignant cells are nonrandomly organized with vascular, hypoxic, stromal, and immune components across GBM tissues [57,58]. Within this framework, perivascular, hypoxic, extracellular matrix (ECM)/invasive, and immunosuppressive niches provide distinct yet overlapping vascular, hypoxic, biomechanical, and immune signals and pressures that collectively shape GSC maintenance and state plasticity [59]. Such niche heterogeneity may in turn impose selective pressures that favor GSC persistence and recurrence.

### 5.1. Perivascular Niche and GSC-Vascular Remodeling

GSCs preferentially associate with tumor vasculature, in which vascular and perivascular cellular components, together with locally derived signals, constitute a specialized niche that regulates GSC maintenance and behavior [60,61]. Within this niche, endothelial-derived signals dynamically regulate GSC stemness and phenotype. Glioma cells adjacent to tumor vessels exhibit stronger stemness signatures and respond to endothelial-derived stromal cell-derived factor-1α (SDF-1α) and C-X-C motif chemokine ligand 12 (CXCL12) through C-X-C motif chemokine receptor 4 (CXCR4)-GLI family zinc finger 1 (GLI1) signaling [62]. In contrast, endothelium-derived semaphorin 3G (SEMA3G) inhibits the stemness of GSCs by promoting c-Myc degradation, suggesting that vascular microenvironment signaling can have the opposite effect on the maintenance of GSCs [63]. Endocan derived from the endothelium further activates platelet-derived growth factor receptor alpha (PDGFRA) signaling in patient-derived gliomaspheres, promoting stable phenotypic remodeling and radioresistance, suggesting that vascular microenvironment signaling can shape treatment-adaptive phenotypes in the stem cell-like GBM population in addition to regulating the nature of stem cells [64].

Importantly, this relationship is bidirectional rather than unidirectional. GSCs also promote vascular remodeling through paracrine signaling, among which interferon-induced transmembrane protein 3 (IFITM3) activates Janus kinase 2 (JAK2)/STAT3-dependent basic fibroblast growth factor (bFGF) secretion, thereby promoting endothelial angiogenesis [65]. Mesenchymal GSC-derived extracellular vesicles actively reshape the vascular microenvironment by reprogramming endothelial cells through the transfer of EGFR/epidermal growth factor receptor variant III (EGFRvIII) [66]. In addition to paracrine signaling and vesicle signaling, GSCs can also directly participate in the formation of perivascular regions by generating pericytes derived from GSCs [67]. This mutual remodeling process may further alter signals that regulate the maintenance and adaptation of stem-like states, thereby linking vascular microenvironmental dynamics with GSC state plasticity. At the functional level, the perivascular niche (PVN) can provide a protective reservoir, which is conducive to maintaining treatment tolerance and supporting tumor reproliferation and recurrence [61,68].

### 5.2. Hypoxic Niche and GSC-like State Plasticity

Spatial profiling has revealed that hypoxia contributes to the organization of distinct malignant cell states across GBM tissue [69]. Consistently, a recent spatial multiomic atlas revealed a hypoxic perinecrotic zone with community-curated glioma stem-like cell marker expression (PNZ_ccGSC), providing an anatomical context in which oxygen limitation may dynamically shape GSC-like states [70]. In this spatially restricted hypoxic context, hypoxia-inducible factor (HIF)-dependent responses provide a mechanistic basis through which GSCs adapt to local oxygen limitation [71,72]. Experimentally induced hypoxia and necrosis trigger rapid niche restructuring and GSC enrichment in perinecrotic regions, supporting a role for hypoxic stress in dynamically reshaping the spatial GSC compartment [73]. Together, these findings link spatial oxygen heterogeneity to the spatial organization and adaptive plasticity of GSC-like states.

### 5.3. ECM and Invasive Niches in GSC State Plasticity

The GBM ECM is spatially heterogeneous, generating region-specific adhesive and mechanical cues rather than serving as a passive scaffold [74]. Within this niche, GSCs respond to matrix mechanics through Yes-associated protein 1 (YAP1)-dependent mechanotransduction. Increased matrix stiffness promotes GSC maintenance and radioresistance through YAP1-dependent induction of IQ motif-containing GTPase-activating protein 3 (IQGAP3) and stabilization of SOX2 [75]. More broadly, YAP1/transcriptional coactivator with PDZ-binding motif (TAZ) signaling regulates GSC stemness and cellular plasticity [76]. These findings indicate that ECM-dependent cues can regulate GSC maintenance and stem-like state adaptation. In patient-derived gliomasphere models, distal invasion into the contralateral hemisphere is linked primarily to OPC-like states and occurs predominantly along peri-axonal white-matter tracts, whereas MES-like tumors preferentially exhibit local perivascular invasion. Importantly, cells actively undergoing invasion activate route-specific transcriptional pathways distinct from their baseline tumor core states. The distal-invasion (DiG) pathway is enriched for NPC-like, OPC-like and cell cycle genes, including DLL3, DCX, OLIG1, CADM2 and MKI67, together with the invasion-associated regulator ZEB1, whereas the local invasion (LiG) pathway is enriched for AC-like and OPC-like genes such as CLU, ALCAM and NLGN3 and is associated with ECM-dependent perivascular invasion and TNC deposition [77]. Thus, ECM mechanics and invasive niches may couple GSC-associated state adaptation to anatomically distinct patterns of GBM infiltration, potentially allowing stem-like cells to persist beyond the resection margin and contribute to recurrence.

### 5.4. Immunosuppressive Niche and GSC State Plasticity

Immunosuppressive niches in GBM are spatially heterogeneous rather than uniformly distributed, resulting in region-specific immune contexts that can differentially support stem-like states [78]. In experimentally defined GSC models, stemness and immune suppression are functionally coupled, as intercellular adhesion molecule 1 (ICAM1) sustains GSC self-renewal while promoting an immunosuppressive microenvironment through β-catenin-programmed death-ligand 1 (PD-L1) signaling [79]. Importantly, this spatial organization is directly related to GSC heterogeneity, as classical (CL) and mesenchymal (MES)-type GSC populations are preferentially enriched in reactive immune regions, whereas high CL-MES signatures are associated with increased myeloid and macrophage abundance [35]. These spatially distinct immune contexts may therefore impose differential selective and maintenance pressures on GSC-like states.

Beyond spatial association, GSCs and immune cells reciprocally remodel the immunosuppressive niche. Proinflammatory TAM-derived tumor necrosis factor alpha (TNF-α) sustains GSC proliferation and self-renewal, whereas stressed GSCs secrete TNF-α-induced protein 6 (TNFAIP6) to drive macrophages toward an inflammatory-suppressive state [80]. TAM-derived lactate also enters GSCs through monocarboxylate transporter 1 (MCT1) and promotes KU70 K317 lactylation, promoting DNA repair and stemness while suppressing cyclic GMP-AMP synthase (cGAS)-stimulator of interferon genes (STING) signaling [81]. GSC-intrinsic clock circadian regulator (CLOCK)-tissue factor pathway inhibitor 2 (TFPI2) signaling further couples GSC self-renewal to the accumulation of immunosuppressive microglia [82]. Together, these findings reveal that myeloid-GSC crosstalk occurs through inflammatory, metabolic, and cell-intrinsic pathways, with signaling occurring in both directions. In addition to myeloid cells, infiltrating plasma-cell-derived immunoglobulin G (IgG) activates Fc gamma receptor IIa (FcγRIIA)-protein kinase B (AKT)-mTOR signaling to maintain GSC proliferation and self-renewal [83], whereas oligodendrocyte transcription factor 2 (OLIG2)^+^ glioma stem-like cells suppress C-X-C motif chemokine ligand 10 (CXCL10) expression and thereby restrict cluster of differentiation 8 (CD8)^+^ T-cell infiltration and activation [84]. These interactions collectively establish self-reinforcing immune niches that sustain stem-like and immune-evasive GSC-like states (Figure 2) (Table 1).

## 6. GSC State Plasticity Drives Therapeutic Resistance

### 6.1. GSC Heterogeneity and Emergence of Therapy-Adaptive States

GSC-associated stem-like compartments are functionally heterogeneous in terms of their therapeutic survival capacity, allowing treatment pressure to increase preexisting resistance states rather than eliminate all stem-like cells [87,88]. Recent studies highlight that therapeutic pressure acts as a selective force that reshapes GBM cell states, allowing plastic tumor populations to escape treatment through adaptive state transitions [89]. Hitomi et al. demonstrated that the asymmetric division of GSCs can generate progeny cells with different therapeutic responses. Among them, the coenrichment of EGFR and the p75 neurotrophin receptor (p75NTR) marked a group of cell subpopulations with greater therapeutic resistance, which tended to survive during treatment [90]. However, the treatment of drug resistance is not solely determined by the preexisting drug-resistant population. In addition to the selection of the existing tolerant population, therapeutic stress can also actively induce adaptive GSC-like states, which can achieve reversible survival, maintain the continuity of treatment, and provide a reserve pool for subsequent drug resistance evolution and tumor recurrence. Liau et al. confirmed that targeted therapy can induce GSCs to enter a slow-cycle persistent cell state through adaptive chromatin remodeling, revealing a reversible survival pathway that is the basis of drug tolerance [86]. Consistent with these findings, lineage tracing analysis revealed that the adaptive persistent state might precede stable drug resistance-related alterations, suggesting that phenotypic plasticity and clonal evolution can act together during the adaptive treatment response of stem-like GBM cells [91]. These results support a model in which the formation of a treatment-tolerant GSC-like state is the result of the combined effects of preexisting heterogeneity, treatment selection, and reversible adaptive transitions rather than a single fixed drug resistance mechanism.

GSC-associated stem-like compartments are heterogeneous in their spatial distribution, molecular pathways, and ability to survive therapeutic stress. In the prospective STEMRI trial, metabolically active fluid-attenuated inversion recovery (FLAIR) regions with a choline/N-acetyl-aspartate index greater than 2 (CNI+) were enriched in GSCs and in stemness, DNA repair, adhesion/migration, and mitochondrial bioenergy pathways, indicating that treatment-naive GBM already contains spatially distinct GSC-enriched reservoirs with properties relevant to therapeutic persistence [92]. Therapeutic pressure can subsequently reshape this preexisting heterogeneity rather than simply eliminate stem-like populations. In patient-derived gliomaspheres containing AC-like, OPC-like, NPC-like, and MES-like states, chemoradiation and vascular cues promote the transition toward a vessel co-opting and resistant cell (VC-Resist) state characterized by resistance to DNA damage, vessel co-option, and senescence/stemness pathways [93]. Thus, therapy can both select cells from a heterogeneous pretreatment population and actively generate adaptive states with distinct survival properties. At the tumor level, longitudinal proteogenomic analysis of 123 matched primary and recurrent GBMs revealed marked changes in cellular pathways during disease evolution, with highly proliferative pathways at diagnosis replaced by neuronal transition and synaptogenic pathways at recurrence [94]. Together, these findings indicate that GSC heterogeneity provides a substrate for differential therapeutic survival, whereas treatment-induced state remodeling further alters the residual stem-like compartment and contributes to the evolving cellular composition of recurrent GBM.

### 6.2. Treatment-Specific GSC Adaptation Pathways

#### 6.2.1. GSC Adaptation to Radiotherapy

In addition to screening out preexisting drug-resistant groups, radiotherapy can also actively induce surviving glioma cells to enter an adaptive state. Radiation-induced cell plasticity promotes cell dedifferentiation or lineage transformation, thereby reshaping the hierarchical structure of tumor cells after treatment [95]. These treatment-induced plastic states can subsequently gradually acquire more stable drug resistance-related characteristics. Repeated radiation stress can induce an adaptive mesenchymal, stem cell-related state characterized by the upregulation of N-cadherin expression, thereby promoting the development of radioresistance [85]. At the molecular level, specific stress response pathways further support the survival of radiation-adapted GSCs. The phosphorylation stability of inosine monophosphate dehydrogenase 2 (IMPDH2) mediated by heat shock protein family A (Hsp70) member 6 (HSPA6) promotes the biosynthesis of guanosine triphosphate (GTP), thereby supporting the survival and maintenance of radiation-resistant GSCs after radiation [96]. These adaptive pathways are reinforced by the inherent DNA damage response ability of GSCs, which enables efficient repair of radiation-induced damage and, in turn, promotes the development of radiation resistance [34].

#### 6.2.2. GSC Adaptation to Chemotherapy

After TMZ exposure, a subset of GBM cells enter senescence and subsequently acquire stem-like properties through HIF-1α/HIF-2α-mediated senescence-associated reprogramming [40]. In addition to treatment-induced acquisition of stemness, intrinsic stemness pathways also contribute to TMZ adaptation. KH RNA binding domain-containing, signal transduction associated 3 (KHDRBS3), containing the KH domain, promotes self-renewal and TMZ resistance in GBM stem-like cell models, linking stem-like properties to chemotherapy tolerance [97]. In addition, GSCs can promote TMZ resistance through intercellular communication. Among them, activating the expression of ATP-binding cassette subfamily B member 4 (ABCB4) driven by activating transcription factor 3 (ATF3) mediates the exosome transfer of drug resistance-related factors, thereby promoting the persistence of tumors after treatment [98]. In addition to TMZ, other cytotoxic agents have been evaluated in the context of stem cell-directed chemotherapy in GBM. In a randomized trial of recurrent GBM, a functional assay measuring the responses of patient-derived cancer stem cells (CSCs) and bulk tumor cells to agents such as carboplatin, carmustine, etoposide, irinotecan, lomustine, procarbazine, and vincristine was used to guide treatment selection, with assay-guided chemotherapy increasing progression-free survival and overall survival compared with physician-selected therapy [99]. These findings indicate that chemotherapy responses in stem-like GBM populations are not restricted to TMZ and that non-TMZ cytotoxic agents may exhibit differential activity across GSCs and bulk-tumor compartments, whereas chemotherapy-surviving quiescent GSCs can provide a reservoir for subsequent tumor regrowth.

#### 6.2.3. Adaptation to Targeted Therapy in GBM and GSC Models

Unlike traditional cytotoxic therapies, targeted therapy exerts selective pressure on specific carcinogenic dependencies and may promote adaptive signaling remodeling in surviving GBM cells, including those in stem-like populations. MET inhibitor-resistant GBM models exhibit activation of compensatory receptor tyrosine kinase (RTK) networks, indicating that pathway switching can support treatment efficacy [100]. Consistent with this, the blockage of EGFR/MET signaling is regulated by sprouty RTK signaling antagonist 2 (SPRY2), which induces the nuclear factor κB (NF-κB)–fibroblast growth factor receptor (FGFR)–extracellular signal-regulated kinase (ERK) bypass axis, thereby restoring downstream survival signaling and promoting resistance to RTK-targeted therapy [101]. This finding indicates that GBM resistance to targeted therapy can arise from reversible signaling adaptation rather than from a fixed stem-cell state alone. Beyond bypass activation, sustained reliance on RTK signaling pathways contributes to therapeutic tolerance in GSCs, as FGFR inhibition reduces GSC maintenance and increases sensitivity to tumor treatment, highlighting adaptive signaling dependencies in therapy-persistent GSCs as exploitable vulnerabilities [102].

## 7. GSC-Associated States and Recurrence

### 7.1. Residual Stem-like Reservoirs Contribute to Recurrence

Although current therapies eliminate a large proportion of tumor cells, residual tumor-propagating and stem-like populations may persist after treatment. SOX2- and CD44-positive GSC-associated stem-like cells remain detectable within postoperative residual tumor sites, providing direct evidence that stem-like populations persist after surgical debulking [103]. Residual stem-like populations should not be viewed as passive remnants. At the tumor level, the posttreatment minimal residual disease state represents a dynamic evolutionary stage in which surviving populations retain or acquire fitness advantages that enable subsequent tumor regrowth [104]. A recent induced-recurrence model using patient-derived glioma-initiating cells (GICs) reproduced an initial treatment response followed by tumor regrowth and patient-specific recurrent GIC phenotypes, further supporting the dynamic evolution of stem/initiating populations across recurrences [105]. Consistently, single-cell profiling combined with patient-derived xenograft models revealed that quiescent CSCs constitute a functionally distinct population that persists after treatment and becomes enriched during recurrence [106]. The microenvironmental context may further shape this residual reservoir, as rare Thy-1 cell surface antigen (THY1)-positive mesenchymal/stem-like populations associated with perivascular regions in treatment-naive GBM are markedly expanded in recurrent tumors following chemoradiotherapy [107]. These findings support a model in which residual stem-like populations, including functionally defined GIC and quiescent CSC compartments, constitute a dynamic reservoir capable of contributing to posttreatment recurrence.

### 7.2. Remodeling of GSC-Associated States in Recurrent Tumors

Upon recurrence, residual GSC-associated populations not only restore the original tumor architecture but also undergo further state remodeling. Single-cell analysis of matched primary and recurrent GBM specimens revealed the emergence of relapse-related subpopulations, indicating that the tumor cell state underwent significant reorganization during the recurrence process [108]. Within the stem-like compartment, this remodeling includes expansion of a CA9-high MES-like GSC subpopulation in recurrent GBM, which exhibits prominent stemness characteristics [109]. More broadly, compared with primary tumors, recurrent tumors exhibit enrichment of GSC-associated stemness pathways, as well as different phenotypic cell clusters, accompanied by increased expression of stem-related factors, including SOX2, POU class 3 homeobox 2 (POU3F2), OLIG2, and NOTCH1 [110]. Longitudinal analysis of circulating extracellular vesicles further revealed dynamic changes in GSC-associated marker profiles during treatment and disease progression, providing patient-level evidence of evolving GSC-associated marker profiles [111]. Consistent with these state-level changes, compared with untreated tumors, GSC-derived tumors that regrew after irradiation exhibited altered spatial growth patterns, reduced transcriptional heterogeneity, and a distinct gene expression profile, extending recurrence-associated remodeling to the spatial and transcriptional organization of the tumor [112]. At the molecular level, GSC lines derived from second-surgery tumors exhibited a different distribution of pathway-level alterations from those derived at initial surgery, including greater representation of RTK and mitogen-activated protein kinase (MAPK) signaling alterations [113]. Together, these findings indicate that recurrent GBM is not a simple restoration of the primary tumor but is progressively reconstructed through the redistribution of GSC-associated states, the reinforcement of stemness pathways, and molecular divergence among recurrent, experimentally defined GSC populations (Figure 3).

## 8. Therapeutic Targeting of GSC Plasticity

Given the continuous remodeling of GSC-like states during therapeutic adaptation and recurrence, a range of experimental strategies have been proposed to therapeutically manipulate the processes that enable state remodeling. Importantly, most approaches discussed in this section remain at the preclinical proof-of-concept stage and should be distinguished from clinically established GBM treatment modalities. Accordingly, these preclinical strategies can be organized into three complementary levels: redirecting or destabilizing existing GSC-like states, broadening therapeutic coverage across heterogeneous GSC populations and protective niches, and intercepting newly induced adaptive states during treatment. Together, these approaches aim to constrain both preexisting and therapy-emergent routes of stem-like cell escape.

### 8.1. Targeting Stem-like State Transitions and GSC Maintenance

Preclinical studies suggest that, rather than simply eliminating experimentally defined GSC populations, therapeutic manipulation of plasticity may destabilize the stem-like state itself. For example, kinesin family member 20A (KIF20A) inhibition drives GBM stem/progenitor cells out of the cell cycle and toward a postmitotic differentiated state, thereby suppressing their proliferative capacity and highlighting differentiation therapy as a strategy to disrupt stem-like tumor populations [114]. A complementary strategy is to release the intrinsic differentiation blockade. Dehydrogenase/reductase 13 (DHRS13) depletion promotes the retinoic acid-dependent differentiation of glioma stem-like cells and subsequently induces mitochondrial reactive oxygen species (ROS)-driven mitophagy and cell death, reducing tumor initiation and progression [115]. Together, these findings indicate that forcing stem-like cells away from proliferative and undifferentiated states may reduce their tumor-propagating capacity.

Beyond differentiation induction, regulators of GSC-like state transitions and GSC maintenance may provide additional therapeutic options. The inhibition of lysine acetyltransferase 5 (KAT5) promotes the emergence of quiescent G0-like neurodevelopmental subpopulations while reducing GSC self-renewal, tumor progression, and invasion [116]. Similarly, disruption of GSC-maintaining dependencies provides another means of destabilizing the stem-like state. Targeting chaperone-mediated autophagy (CMA) through the mammalian STE20-like protein kinase 4 (MST4)-lysosome-associated membrane protein 2A (LAMP2A) axis suppresses GSC proliferation and self-renewal by limiting mechanistic targeting of rapamycin complex 1 (mTORC1) activation while also promoting antitumor immunity, revealing CMA as a therapeutically actionable dependency of GSCs [117]. Collectively, these studies provide preclinical proof of concept that stem-like states and experimentally defined GSC populations can be perturbed by inducing differentiation, redirecting cellular states, or disrupting stemness-supporting dependencies. However, these approaches have not yet been clinically validated as GSC-directed therapies in GBM, and plasticity may allow surviving cells to shift toward alternative states, providing a rationale for broader multistate or combination strategies.

### 8.2. Overcoming GSC Heterogeneity and Niche Barriers

Preclinical strategies have also attempted to limit state-dependent therapeutic escape by targeting GSC heterogeneity and niche-associated barriers. Mesenchyme homeobox 2 (MEOX2) and serglycin (SRGN) represent the selective weaknesses of CL and MES GSCs, respectively. The combined targeting of the two has shown greater antitumor efficacy both in vivo and in vitro [35]. As a surface antigen enriched in GSCs, cluster of differentiation 97 (CD97) supports tumorigenesis through the mechanistic target of rapamycin complex 2 (mTORC2) signaling pathway. Moreover, chimeric antigen receptor (CAR) T-helper 9 (Th9) cells targeting CD97 can effectively eliminate GSCs and prolong survival in vivo [118]. Similarly, protein tyrosine phosphatase receptor type Z1 (PTPRZ1)-specific T-cell receptor-engineered T (TCR-T) cells preferentially target GSCs and AC-like tumor cells and exhibit antitumor activity in experimental GBM models, supporting the use of stem-associated antigens as targets for adoptive T-cell therapy [119]. Although these studies support the feasibility of targeting GSC-associated antigens, the CD97-directed CAR-Th9- and PTPRZ1-specific TCR-T-cell approaches described here were evaluated in experimental GBM models and should not be interpreted as clinically established GSC-directed therapies. Beyond target selection, effective GSC-directed therapy also requires access to stem-like cells within protected niches. Biomimetic monocyte membrane-coated nanoparticle (MoNP)-mediated delivery of verteporfin increases therapeutic access to perivascular GSCs and suppresses their growth and invasion, providing preclinical evidence that overcoming barriers to niche-associated delivery may increase access to protected GSCs [120]. Thus, overcoming GSC heterogeneity may require broadening both molecular target coverage and spatial therapeutic access across diverse GSC populations and GSC-like states.

### 8.3. Blocking Therapy-Induced Adaptive Plasticity

In addition to targeting existing GSC-like states, preclinical studies suggest that treatment-induced adaptive remodeling may create transient vulnerabilities that could be therapeutically intercepted. Therapy-induced state transitions can expose glioma stem-like cells to transient vulnerabilities, as targeting treatment-induced transcriptional pathways with small interfering RNA (siRNA) or a second drug promotes the elimination of surviving patient-derived glioma stem-like cells and disrupts acquired resistance [121]. At the epigenetic level, targeting bromodomain-containing protein 2 (BRD2) suppresses mesenchymal-state plasticity in GBM and increases radiation sensitivity, including in GBM stem cell models, indicating that therapy-resistant state transitions can be therapeutically intercepted [122]. Similarly, pharmacological targeting of the moesin (MSN)-phosphoinositide 3-kinase (PI3K)/mTOR axis with GNE-317 reversed proneural-to-mesenchymal transition in GSCs and restored sensitivity to radiotherapy and TMZ [58]. Beyond mesenchymal remodeling, therapy-induced plasticity can also generate quiescent states, as TMZ-induced SRY-box transcription factor 10 (SOX10) suppression drives GBM cells toward a quiescent NSC-like state, whereas Notch inhibition reduces this therapy-associated population, further indicating that adaptive state transitions can be therapeutically redirected [123]. Together, these studies provide experimental proof of concept that therapy-induced state remodeling can be intercepted. BRD2 inhibition, pharmacological targeting of the MSN-PI3K/mTOR axis with GNE-317, and redirection of SOX10-associated quiescent states therefore represent preclinical strategies that require further validation before their clinical relevance can be established.

## 9. Discussion

GSC state plasticity provides a framework linking therapeutic adaptation to resistance and recurrence in GBM. However, the proneural-to-mesenchymal transition remains the best characterized transcriptional shift, whereas direct interconversion among all four major states remains unproven.

Despite the abovementioned progress, some fundamental issues remain unresolved. First, owing to the lack of universal markers and the ability of stem cell-like cells to transition across phenotypic states, the operational identification of GSC populations remains context-dependent and complex [29]. Second, traditional preclinical models often struggle to fully reproduce the complex cellular and molecular dynamics of GBM, leading to a translational gap between preclinical research results and clinical outcomes [124]. Third, integrating single-cell and spatial transcriptomics findings into a clinically actionable framework remains a major challenge [57]. Fourth, plasticity remains a difficult problem for persistent targeting because existing therapeutic strategies have not achieved lasting clinical benefits, and drug-tolerant persister cells can survive through reversible nongenetic adaptive mechanisms [89,125]. Finally, the therapeutic window for intercepting adaptive states remains a clinically testable hypothesis rather than a validated interval [126].

There are still many obstacles in the transformation of these concepts into clinical applications. The blood–brain barrier and heterogeneous blood–tumor barrier prevent most drugs from reaching invasive tumor areas [126]. Intratumoral heterogeneity further limits the efficacy of single-target treatment strategies. Residual cell populations can convert transcription-dependent pathways and activate compensatory pathways, thereby driving the development of drug resistance [125,127]. The contrast between encouraging preclinical findings and limited clinical efficacy is particularly evident for immune-based therapies. In a phase III clinical trial of GBM, immune checkpoint inhibitors failed to increase the survival of patients [128,129,130]. Clinical trials of CAR-T cells have reported acceptable safety; however, the responses are transient and variable, and implantation and persistence are limited [131]. An immunosuppressive microenvironment and antigen heterogeneity remain the main obstacles to achieving durable therapeutic effects [132]. Moreover, most candidate targets have been identified in experimental models that incompletely reproduce human disease [132]. Biomarkers that reliably report GSC-like states in individual patients are also lacking, although liquid biopsy platforms offer a possible path forward [111]. Without robust biomarkers of GSC-like states together with functional validation, patient stratification and the clinical implementation of GSC-directed therapies remain challenging [133].

Several emerging technologies are now helping close these gaps. Single-cell multiomics and spatial transcriptomics can map GSC-like states within their native niches and reveal how these states reorganize from diagnosis to recurrence [134]. Genome-wide clustered regularly interspaced short palindromic repeats (CRISPR) screens reveal context-specific dependencies of therapy-adapted glioma cells [135]. Patient-derived organoids retain key features of the parental tumor and can act as real-time avatars for evaluating CAR-T-cell therapy [136]. Liquid biopsy based on circulating extracellular vesicles enables longitudinal monitoring of tumor evolution and may capture stemness-associated phenotypes at recurrence [137,138]. The application of machine learning to multiomics data can stratify patients and identify therapeutic targets [139]. New delivery technologies, including optical regulation of the blood-brain tumor barrier and focused ultrasound-mediated barrier opening, are also constantly advancing [140,141]. In summary, these tools are driving the field from static description to dynamic, patient-specific measurement of GSC state plasticity.

To achieve durable control of GBM, it is highly likely that a combined strategy will be needed to limit the remodeling of GSC-like state composition, expand the treatment coverage for heterogeneous populations and protective microenvironments, and intercept treatment-induced adaptive transitions. Translating this concept into clinical practice will rely on status-reported biomarkers and longitudinal monitoring to guide patient stratification and treatment timing. Therefore, emerging technologies may be able to transform the state plasticity of GSCs from a biological framework into a clinically testable therapeutic framework.

## 10. Conclusions

Collectively, these findings suggest that GSC state plasticity provides a unifying framework for understanding how GBM adapts to environmental and therapeutic pressures, persists after treatment, and ultimately recurs. Rather than representing a fixed cellular population, stem-like phenotypes are dynamically shaped by transcriptional state transitions, metabolic adaptation, reversible quiescence, and reciprocal interactions with vascular, hypoxic, extracellular matrix, and immune niches. These interconnected processes enable therapeutic pressure not only to select preexisting tolerant populations but also to generate adaptive states that survive treatment and contribute to the reconstruction of recurrent tumors. Accordingly, targeting a single marker, pathway, or cellular state is unlikely to fully address the adaptive capacity of GBM. More durable therapeutic control may instead require integrated strategies that constrain state transitions, target complementary vulnerabilities across heterogeneous stem-like populations, disrupt protective niche interactions, and intercept treatment-induced adaptation. Translating this framework into clinical practice will depend on increased longitudinal monitoring of GSC-like states and validation of plasticity-directed strategies in clinically relevant models and patients.

## Figures and Tables

**Figure 1:**
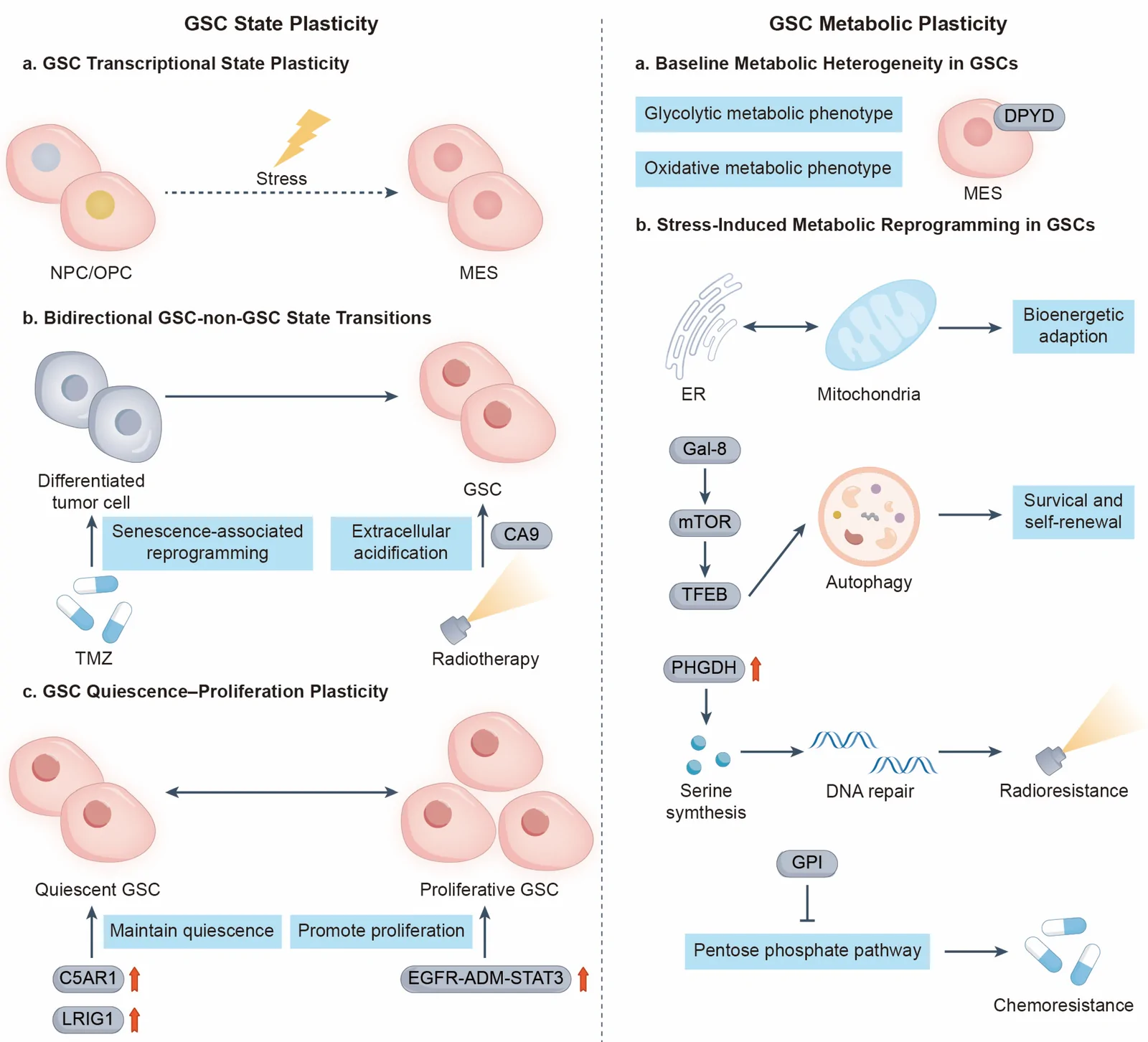
**GSC-associated state plasticity and metabolic reprogramming in glioblastoma.** GSC State Plasticity. (**a**). GSC-like transcriptional state plasticity. The schematic highlights the well-characterized stress-associated transition from proneural-associated NPC-like/OPC-like states toward a MES-like state, with microenvironmental and therapeutic stress promoting the proneural-to-mesenchymal transition. It does not imply direct interconversion among all four major transcriptional states, which remains unproven. (**b**). Bidirectional transitions between stem-like and differentiated tumor states. Differentiated tumor cells can acquire stem-like properties in response to radiotherapy (via CA9-dependent extracellular acidification) or temozolomide (TMZ, via senescence-associated reprogramming). Conversely, cells occupying stem-like states may exit these states to adopt distinct fates, including neuronal-like transdifferentiation or vascular lineage contributions. (**c**). Quiescence–proliferation plasticity. Quiescence represents a reversible state within experimentally defined GSC populations that supports stress tolerance and therapeutic persistence. C5AR1 signalling favours G_0_ maintenance, whereas EGFR–ADM–STAT3 signalling promotes self-renewal and proliferation. LRIG1 regulates the balance between these opposing states. Following chemotherapy, surviving F3^+^ (tissue factor-positive) quiescent GSCs become activated and transition toward proliferation, contributing to tumor expansion and recurrence. GSC Metabolic Plasticity. (**a**). Baseline metabolic heterogeneity in GSC models. Patient-derived GSC models display distinct glycolytic and oxidative metabolic phenotypes, and DPYD expression correlates with MES-like features, indicating state-dependent metabolic configurations. (**b**). Stress-induced metabolic reprogramming. Under hypoxia, GSCs activate Gal-8-mTOR-TFEB-dependent autophagy to sustain survival. Irradiation redirects glycolytic flux toward PHGDH-dependent serine synthesis to support DNA repair, while TMZ-induced GPI inactivation shifts glucose flux into the pentose phosphate pathway, promoting chemoresistance. ER–mitochondrial communication and bioenergetic adaptation further support GSC fitness under nutrient stress.

**Figure 2:**
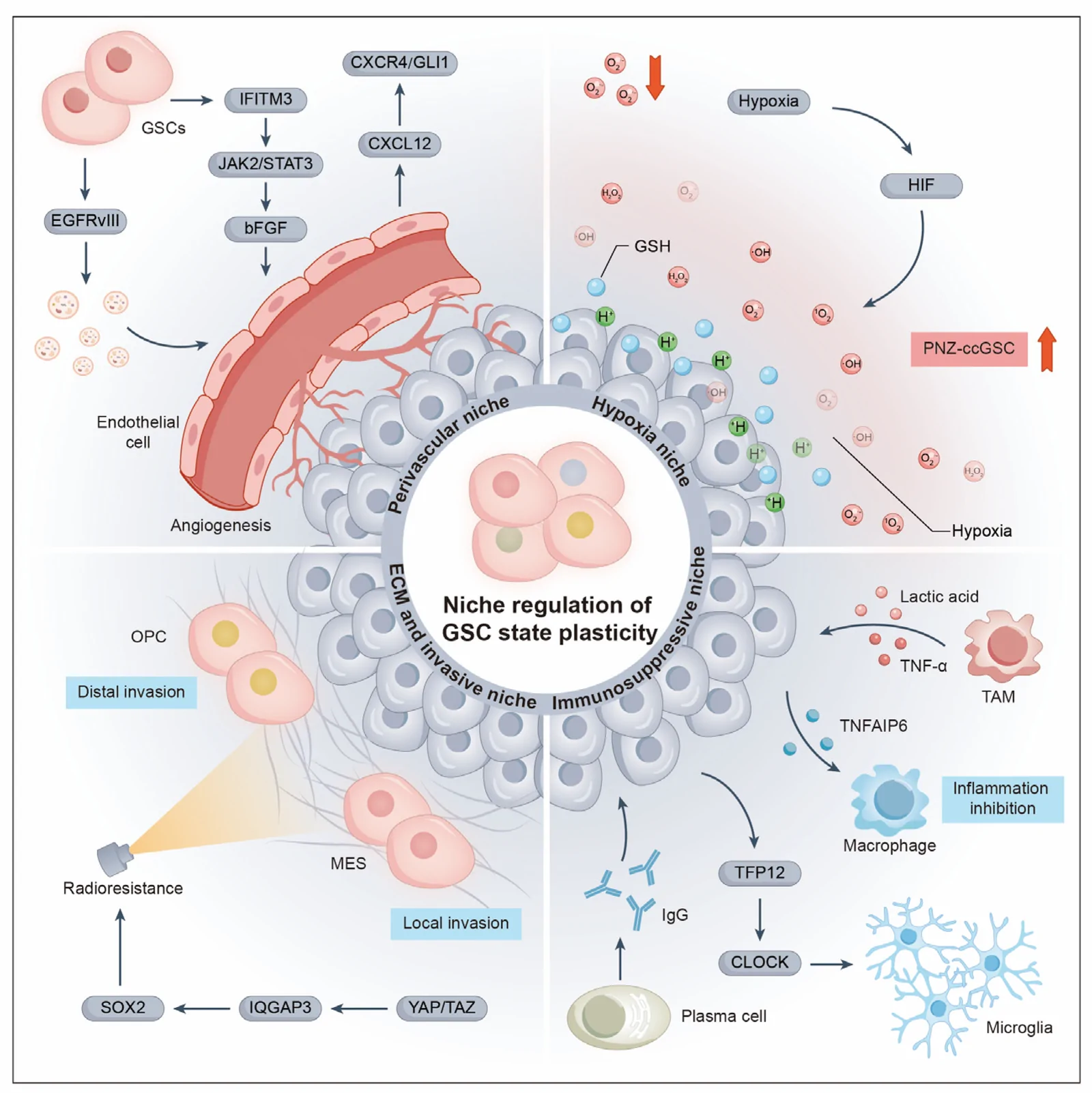
**Niche regulation of GSC-associated state plasticity. Spatially and functionally distinct niches collectively shape the maintenance and adaptive plasticity of GSC-associated stem-like states**. In the **perivascular niche**, endothelial-derived CXCL12-CXCR4-GLI1 signalling sustains GSC stemness, whereas SEMA3G opposes this effect by promoting c-Myc degradation. GSCs reciprocally remodel the vasculature via IFITM3–JAK2–STAT3-driven bFGF secretion and EGFRvIII-containing extracellular vesicles, which promote angiogenesis and can directly generate pericytes. In the **hypoxic niche**, HIF-dependent responses and perinecrotic zones (PNZ_ccGSC) dynamically shape GSC-like states. In the ECM/invasive niche, increased matrix stiffness activates YAP/TAZ–IQGAP3–SOX2 mechanotransduction, thereby supporting stemness and radioresistance; OPC-like and MES-like states preferentially associate with distal and local invasion programs, respectively. In the **immunosuppressive niche**, TAM-derived TNF-α sustains GSC proliferation, while GSC-derived TNFAIP6 and CLOCK–TFPI2 signalling promote inflammatory-suppressive macrophage and microglia accumulation. TAM-derived lactate enters GSCs via MCT1 to enhance DNA repair and suppress cGAS–STING signalling. Infiltrating plasma cell-derived IgG activates FcγRIIA-AKT-mTOR signalling, and OLIG2+ glioma stem-like cells suppress CXCL10 expression and are associated with reduced CD8+ T-cell infiltration, collectively contributing to a self-reinforcing immune-evasive niche.

**Figure 3:**
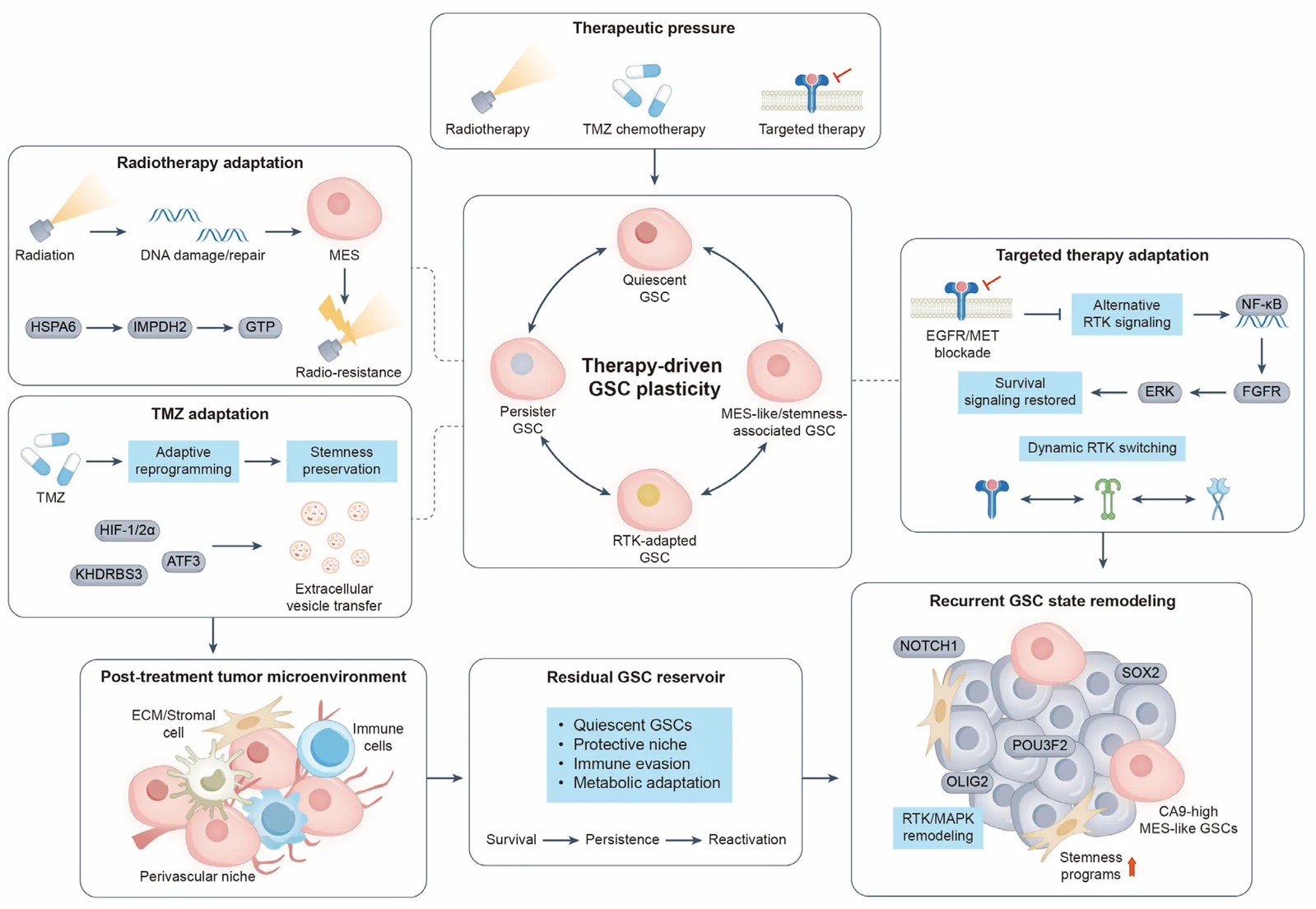
Therapy-driven remodeling of GSC-associated states and recurrence. Therapeutic pressure can reshape GSC-associated states through selection of pre-existing tolerant populations and induction of adaptive programs. Radiotherapy promotes DNA damage repair and drives a mesenchymal-like, stemness-associated state with N-cadherin upregulation and HSPA6–IMPDH2-mediated GTP biosynthesis. TMZ induces adaptive reprogramming via HIF-1α/HIF-2α-mediated senescence-associated pathways and ATF3-driven ABCB4 exosomal transfer, while preserving a quiescent GSC-like reservoir that may reactivate after treatment. Targeted therapy, including EGFR/MET blockade, can activate bypass signaling through NF-κB–FGFR–ERK pathways and alternative RTK networks, sustaining survival signalling and therapeutic persistence. Post-treatment, persister stem-like cells, therapy-adapted MES-like populations, and quiescent cells reside in protective niches (perivascular, immune-evasive and metabolically remodelled), forming residual reservoirs. During recurrence, these populations undergo further state remodeling, with expansion of CA9-high MES-like GSC-like populations, upregulation of stemness programs (SOX2, POU3F2, OLIG2, NOTCH1) and RTK/MAPK pathway divergence, collectively contributing to progressive tumor regrowth distinct from the primary lesion.

**Table 1:** Major dimensions of GSC plasticity.

Dimension	Key State or Transition	Representative Molecules and Pathways	Functional Consequences	Ref.
Transcriptional state plasticity	Proportional remodeling of NPC-like, OPC-like, AC-like, and MES-like states	PDGFRA, EGFR, CDK4, NF1; MEOX2-NOTCH, SRGN-NF-κB	Intratumoral heterogeneity; therapy resistance	[8,35]
	PN → MES transition	MSN-PI3K/mTOR	Stemness; chemoradiotherapy resistance	[58]
	RT-induced resistant state	IGF1-N-cad-β-catenin-CLU	Stemness; radioresistance	[85]
Stem-like ↔ differentiated-state plasticity	TMZ-treated GBM cell → GSC-like state	HIF-1α/HIF-2α-SASP	Stemness; invasion; TMZ resistance	[40]
	Irradiated differentiated GBM cell → GSC-like state	OXPHOS-CA9-acidosis	Stemness; radioresistance	[39]
	GSC → neuron-like state	SYN3-NRG3-JAG1/NOTCH1	Reduced stemness; tumor suppression	[42]
	GSC → GDEC	WTAP-ROR1-WNT5A	Vasculogenesis; TMZ resistance	[43]
Quiescence-proliferation plasticity	G0-phase maintenance	C3-C5AR1-JAK2/STAT3/STAT5	Stemness; metabolic drug resistance	[45]
	Quiescent GSC → proliferative GSC	F3 (CD142); GS-118 signature	Chemoresistance; recurrence	[47]
	Quiescent GSC ↔ proliferative GSC	LRIG1-EGFR/BMP	Tumor growth; therapy persistence	[48]
	Proliferative GSC ↔ slow-cycling persister GSC	Notch-KDM6A/B-H3K27me3	Drug tolerance; relapse	[86]
Metabolic plasticity	Glycolytic and oxidative phenotypes	Glycolysis-TCA cycle	Metabolic heterogeneity; cell-intrinsic persistence	[49]
	Glycolysis ↔ OXPHOS adaptation	ERO1α-MAM-Ca^2+^	Metabolic flexibility; GSC aggressiveness	[52]
	Glycolysis → serine synthesis	ATM-PGK1-PHGDH	DNA repair; radioresistance	[55]
	Glycolysis → PPP	ATM-PLK1-GPI	Redox adaptation; TMZ resistance	[56]
Niche-regulated state plasticity	Perivascular induction of GSC stemness	SDF-1α-CXCR4-AKT/NF-κB-GLI1	Stemness; tumor progression	[62]
	GSC stemness suppression	SEMA3G-NRP2/PLXNA1-WWP2-c-Myc	Reduced stemness; tumor suppression	[63]
	Perivascular phenotype remodeling	Endocan-PDGFRA-Myc	Spatial identity; radioresistance	[64]
	Hypoxic/perinecrotic remodeling of GSC-associated states	Necrosis-TAM-GSC axis	Stem-like/GSC enrichment; tumor expansion	[70,73]
	Stiff ECM → GSC maintenance	YAP1/TEAD-IQGAP3-SOX2	Stemness; radioresistance	[75]
	Immunosuppressive niche support of GSC maintenance	ICAM1-LRP6-β-catenin-PD-L1	Stemness; immune evasion	[79]

Abbreviations: AC: Astrocyte-like; AKT: Protein kinase B; ATM: Ataxia telangiectasia mutated; BMP: Bone morphogenetic protein; C3: Complement component 3; C5AR1: Complement C5a receptor 1; CA9: Carbonic anhydrase IX; CD142: Cluster of differentiation 142; CDK4: Cyclin-dependent kinase 4; CLU: Clusterin; CXCR4: C-X-C motif chemokine receptor 4; ECM: Extracellular matrix; EGFR: Epidermal growth factor receptor; ERO1α: Endoplasmic reticulum oxidoreductase 1 alpha; F3: Coagulation factor III; GDEC: Glioma-derived endothelial cell; GLI1: GLI family zinc finger 1; GPI: Glucose-6-phosphate isomerase; GSC: Glioblastoma stem cell; H3K27me3: Histone H3 lysine 27 trimethylation; HIF-1α: Hypoxia-inducible factor 1 alpha; HIF-2α: Hypoxia-inducible factor 2 alpha; ICAM1: Intercellular adhesion molecule 1; IGF1: Insulin-like growth factor 1; IQGAP3: IQ motif-containing GTPase-activating protein 3; JAG1: Jagged canonical Notch ligand 1; JAK2: Janus kinase 2; KDM6A/B: Lysine demethylase 6A/6B; LRIG1: Leucine-rich repeats and immunoglobulin-like domains 1; LRP6: Low-density lipoprotein receptor-related protein 6; MAM: Mitochondria-associated membrane; MEOX2: Mesenchyme homeobox 2; MES: Mesenchymal-like; MSN: Moesin; mTOR: Mechanistic target of rapamycin; N-cad: N-cadherin; NF1: Neurofibromin 1; NF-κB: Nuclear factor kappa B; NPC: Neural progenitor cell-like; NRG3: Neuregulin 3; NRP2: Neuropilin 2; OPC: Oligodendrocyte precursor cell-like; OXPHOS: Oxidative phosphorylation; PD-L1: Programmed death-ligand 1; PDGFRA: Platelet-derived growth factor receptor alpha; PGK1: Phosphoglycerate kinase 1; PHGDH: Phosphoglycerate dehydrogenase; PI3K: Phosphoinositide 3-kinase; PLK1: Polo-like kinase 1; PLXNA1: Plexin A1; PN: Proneural; PPP: Pentose phosphate pathway; ROR1: Receptor tyrosine kinase-like orphan receptor 1; RT: Radiotherapy; SASP: Senescence-associated secretory phenotype; SDF-1α: Stromal cell-derived factor 1 alpha; SEMA3G: Semaphorin 3G; SOX2: SRY-box transcription factor 2; SRGN: Serglycin; STAT3: Signal transducer and activator of transcription 3; STAT5: Signal transducer and activator of transcription 5; SYN3: Synapsin III; TAM: Tumor-associated macrophage; TCA: Tricarboxylic acid; TEAD: TEA domain transcription factor; TMZ: Temozolomide; WNT5A: Wnt family member 5A; WTAP: WT1-associated protein; WWP2: WW domain-containing E3 ubiquitin protein ligase 2; YAP1: Yes-associated protein.“→” indicates a directional transition, whereas “↔” indicates a bidirectional transition.

## Data Availability

No new data were generated or analyzed in this review.
